# Supplementary material for: Global Cardiovascular Risk Profile of Italian Medical Students Assessed by a QR Code Survey. Data from UNIMI HEART SURVEY: Does Studying Medicine Hurt?
Source: J Clin Med. 2021 Mar 24;10(7):1343. doi: 10.3390/jcm10071343 (PMC8037873; doi:10.3390/jcm10071343)
Supplement: Supplementary file 1 [file jcm-10-01343-s001.pdf]

UNIMI-HEART Survey QUESTIONNAIRE

The following questionnaire is addressed EXCLUSIVELY to students of Medical School of Statale of Milan. The data are collected in complete anonymity. The average compilation time is approximately 8 minutes. By filling in the questionnaire, you automatically consent to the use of the collected data. Thanks, for your valuable help.

*Required field

1) GENERAL CHARACTERISTICS

- Age *

- Gender *
  - Male
  - Female
- Faculty campus *
  - Milano statale (Polo San Paolo)
  - Milano statale (Polo Centrale)
  - Milano statale (Polo Sacco/Vialba)
- Year of the course *
  - 1°
  - 2°
  - 3°
  - 4°
  - 5°
  - 6°
  - Repeating student
- Working position *
  - Student
  - Working student
- Commuter (home-university travel time > 1 hour) *
  - No
  - Yes
- Non-resident student*
  - No
  - Yes

2) ANTHROPOMETRIC, CLINICAL AND LIFESTYLE DATA

- Do you think you have a healthy and balanced lifestyle? *
    - o No
    - o Yes
    - o Don't Know

- Weight (Kg) *

- Height (meters) *

- Are you aware of your arterial blood pressure values?*
    - o No
    - o Yes

- If you answered "YES" to the previous question, do your blood pressure values exceed those recommended (120/80 mmHg)?
    - o No
    - o Yes

- Are you aware of your lipidic profile?*
    - o No
    - o Yes

- If you answered "YES" to the previous question, does your total cholesterol exceed 155 mg / dl?
    - o No
    - o Yes

- Are you diabetic? *
    - o No
    - o Yes

- Have your 1st degree relatives (mother and father) had an early cardio / cerebro / vascular event (eg myocardial infarction, stroke) (men <55 years and women <65 years)? *
    - o No
    - o Yes

- Do you think you have a healthy and balanced lifestyle? *
    - o No

- Yes
- Former

- If you answered "YES" to the previous question, how many cigarettes do you smoke per day?
  - 0-10
  - 11-20
  - >20

- If you are a smoker, what type of cigarettes do you smoke?
  - Traditional tobacco cigarettes
  - Vaping system
  - HEAT system/IQOS

- Do you drink alcohol (even occasionally)?*
  - No
  - Yes
- If you answered "YES" to the previous question, do you consume more than 10 drinks per week (if you are a woman) or 15 per week (if you are a man)?
  - No
  - Yes

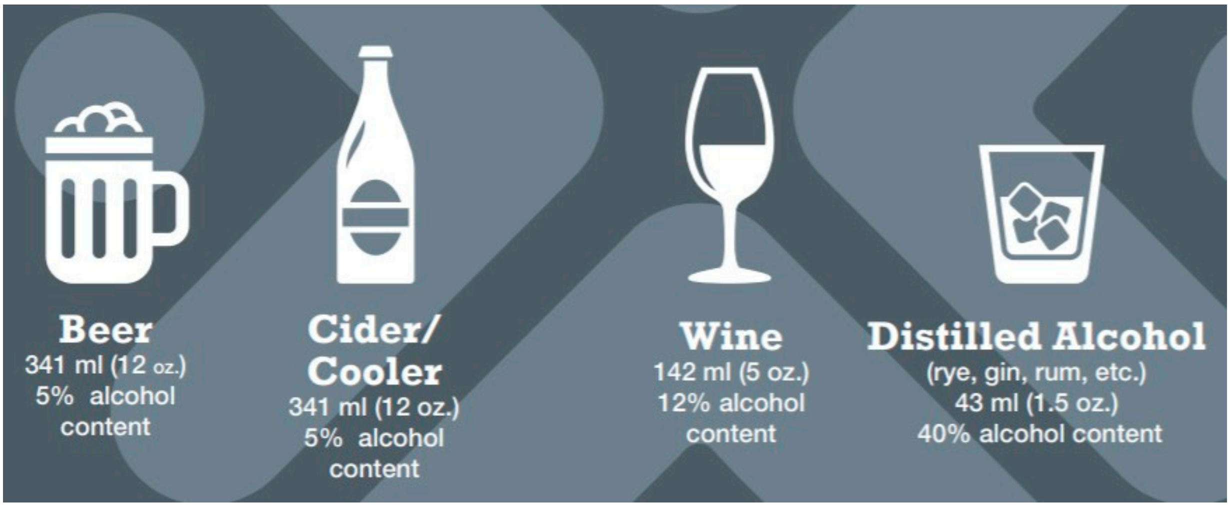

- Do you use drugs / drugs (even occasionally)?
  - No
  - Yes

- How many hours do you sleep on average per night?
  - <6h

- 6-9h
- >9h

- Are you irritable, anxious, having trouble sleeping, because of university problems?*
  - Never
  - Rarely
  - Often
  - Almost always

- Are you irritable, anxious, having trouble sleeping, because of house related problems?*
  - Never
  - Rarely
  - Often
  - Almost always

- Physical activity (choose only one option) *

  Light activity: the heart beats just faster than normal, while the activity is being carried out you can speak without any problems. Moderate activity: the heart beats faster than normal, while the activity is taking place, you can still talk but with a little trouble. Intense activity: the heart beats much faster than normal, while the activity takes place it is difficult to speak.
  - Exercise very rarely or never
  - Light activity but not every week
  - Light activity at least once week
  - Moderate activity but less than 30minutes per day or less than 5 days per week
  - Intense activity, but less than 20 minutes per day or less than 3 days per week
  - Moderate activity at least 30 minutes per day 5 times per week
  - Intense activity at least 20 minutes per day 3 times per week

3) DIET

- Do you use oil as the main cooking fat? *
  - No
  - Yes
- Do you consume at least 4 tablespoons of olive oil per day (including fries, salads, etc.)? *

  tablespoon = 10g
  - No
  - Yes
- Do you consume at least 2 portions of cooked vegetables per day (or 1 portion of raw vegetables)? *

1 serving = 200g

- No
- Yes

- Do you consume at least 3 portions of fruit per day? *

  1 serving = 80g

  - No
  - Yes

- Do you consume more than 1 portion of red meat, processed meat and salami per day? *

  1 serving = 150g

  - No
  - Yes

- Do you consume white meat and poultry more often than red meat (veal, beef, lamb, pork) and processed meat (hamburgers, sausages, cold cuts)?*

  - No
  - Yes

- Do you consume more than 1 serving of butter or margarine per day? *

  serving = 12g

  - No
  - Yes

- Do you consume more than 1 carbonated or sugary drink per day?*

  - No
  - Yes

- Do you drink 7-10 glasses of wine per week? *

  - No
  - Yes

- Do you consume at least 3 portions of legumes per week? *

  1 serving = 150g

  - No
  - Yes

- Do you consume at least 3 portions of fish, crustaceans or shellfishes per week?*

  - No
  - Yes

- Do you consume snacks, biscuits, pastries and industrial sweets up to 3 times a week?*
    - No
    - Yes
- Do you consume at least 3 servings of dried fruit per week? *

  1 serving = 25-30g
    - No
    - Yes
- Do you consume recipes with sautéed base (garlic, onion, leek) at least 2 times a week?*
    - No
    - Yes
- Do you think you are consuming more salt daily than recommended? *

  WHO limit: 5g = about one teaspoon
    - No
    - Yes
    - Don't Know
- Do you think you are adopting a Mediterranean diet? *

  The Mediterranean diet favors cereals, fruit, vegetables, seeds, olive oil, compared to a rarer use of red meats and animal fats (e.g. butter), while suggesting moderate consumption of fish, white meat, legumes, eggs, dairy products , red wine and sweets.
    - No
    - Yes
    - Don't Know

4) DRUGS

- Do you take blood pressure medications?*
    - No
    - Yes
- Do you take medications to reduce cholesterol?*
    - No
    - Yes
- Do you take medications to control diabetes?*
    - No
    - Yes

5) DURING THE YEARS OF MEDICINE STUDIES:

- Have you gained weight?
  - No, steady
  - No, I’ve lost weight
  - Yes
- Have you reduced physical activity?
  - No, steady
  - No, I’ve improved
  - Yes
- Have you started smoking?
  - No
  - No, I stopped
  - Yes
- Do you think you have worsened the quality of your diet?
  - No, steady
  - No, I’ve improved
  - Yes
- Have you increased your alcohol consumption?
  - No, steady
  - No, it’s diminished
  - Yes
- Do you think you are more stressed?
  - No, steady
  - No, less
  - Yes
  - Don’t know
- Do you think you are happier?
  - No, steady
  - No, less
  - Yes
  - Don’t know

- Overall, do you think you have made your lifestyle worse?
    - No, steady
    - No, it’s improved
    - Yes
    - Don’t Know

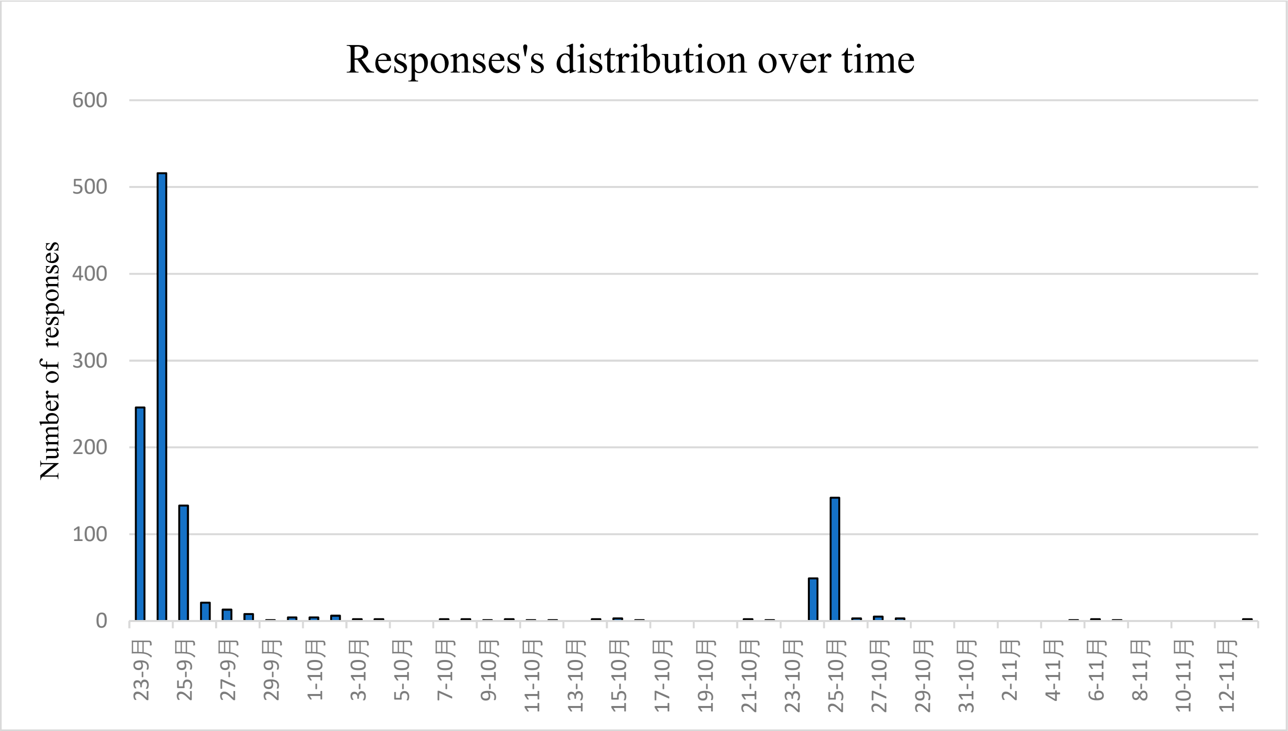

Online Figure 1 *Distribution of responses over time: 1° peak during the academic year's first week and 2° peak after the "recall" flyers delivery.*

N° OF PEOPLE WITH VIRTUOUS PECULIARITIES

Virtuous peculiarities	No.	(%)
18.5 < BMI ≤24.9	942	79.6
Not active smoker	1081	91.4
Not diabetic	1176	99.4
Optimal physical activity	285	24.1
Adequate diet: PREDIMED≥ 10	231	19.5

N° OF PEOPLE WITH ALL THE VIRTUOUS PECULIARITIES

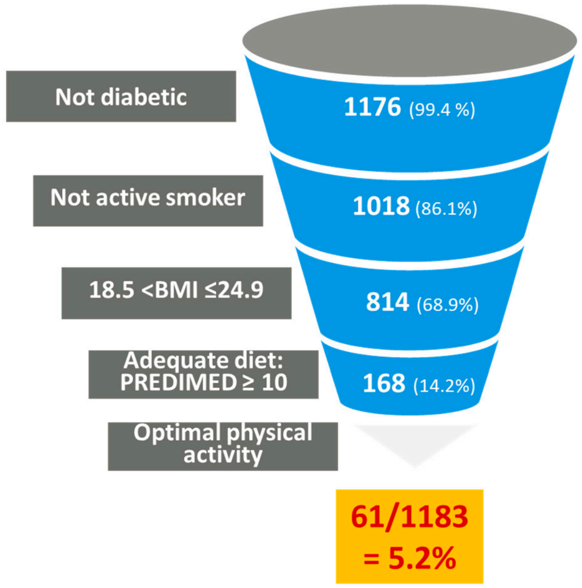

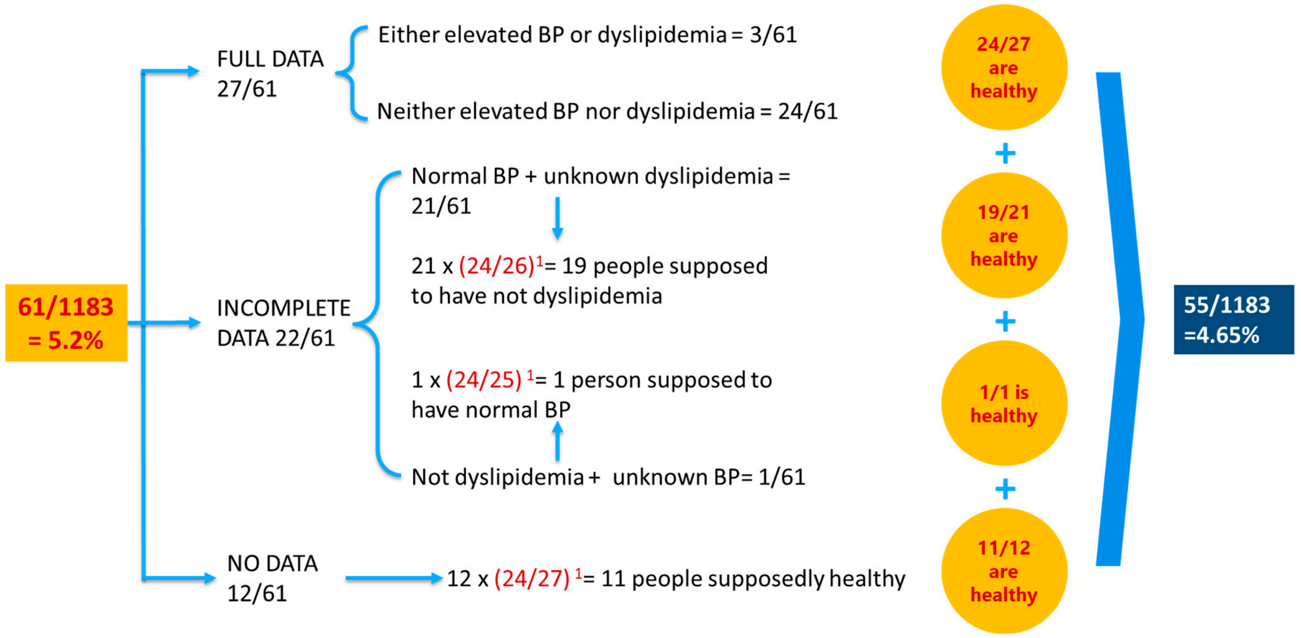

1. The ratio is an estimate derived from the sample for which all the data were available (27 obs), within that subsample it has identified the number of people falling in each category to define the baseline of the ratio itself.

Online Figure 2 *Estimation of IDEAL CARDIOVASCULAR HEALTH*
